# Commentary: Case Report: Massive intrapericardial hematoma following acupuncture therapy

**DOI:** 10.3389/fcvm.2025.1550391

**Published:** 2025-04-30

**Authors:** Sun-Jung Lee, Sung-A Kim, Tae-Hun Kim, Jung Won Kang

**Affiliations:** ^1^ Department of Clinical Korean Medicine, Graduate School, Kyung Hee University, Seoul, Republic of Korea; ^2^ Epidemiology Group, School of Medicine, Medical Sciences and Nutrition, University of Aberdeen, Aberdeen, United Kingdom; ^3^ Korean Medicine Clinical Trial Center, Korean Medicine Hospital, Kyung Hee University, Seoul, Republic of Korea; ^4^ Department of Acupuncture & Moxibustion, College of Korean Medicine, Kyung Hee University, Seoul, Republic of Korea

**Keywords:** acupuncture, intrapericardial hematoma, hemopericardium, cardiac tamponade, STRICTA

A Commentary on Case Report: Massive intrapericardial hematoma following acupuncture therapy By Chen W, Gao X, Li H (2024). Front. Cardiovasc. Med. 11:1433945. doi: 10.3389/fcvm.2024.1433945

Chen et al. (1) reported a case involving an acupuncture-related intrapericardial hematoma with subsequent cardiac tamponade. A 69-year-old female patient developed syncope just 10 min after needle insertion below the xiphoid process. Eight hours later, she was transferred to the hospital with complaints of severe chest pain, nausea, and vomiting. Hemorrhagic effusion in the intrapericardial region was discovered by echocardiography, computed tomography (CT) scan, and pericardiocentesis. She was diagnosed with traumatic hemopericardium, following which she underwent pericardial drainage, which stabilized her condition. The authors mentioned that a relationship could exist between acupuncture and cardiac injury, underscoring the need for careful assessment and prompt management. However, several aspects of this report deserve further discussion from the point of view of acupuncture experts.

First, it is imperative to consider the direction and depth of acupuncture needling to discuss the possibility of heart penetration from the subxiphoid process. Typically, the apex, the lowest part of the heart, is located at the fifth intercostal space, while the sternum is attached to the first to seventh ribs (2). Therefore, unless unique conditions such as advanced age, cardiomegaly, or anomalous physical characteristics are present, the heart rarely extends below the xiphoid process anatomically. According to the acupuncture textbook that the authors referred to, it is recommended to insert the filiform needle directly 0.3–0.6 in deep, or obliquely downward 0.5–1 in deep at the Jiuwei (CV15) point (3) mentioned in this report, but in this manner, penetrating the heart does not seem plausible (4, 5). Only if the needle was inserted at an upward angle at a depth that could reach the heart, could it potentially pierce the heart. In the process of subxiphoid pericardiocentesis, the needle insertion site is generally situated between the xiphisternum and the left costal margin, with the needle angled upward at 15–30° (6). But unlike Western medical treatments like this, acupuncture treatment rarely targets the solid organs like the heart directly but just stimulates nearby or distant acupuncture points on the surface of the body. Thus, without this detailed information, its causality can only be evaluated as “unassessable” according to the modified WHO-UMC causality criteria (7).

Second, this patient case lacks a comprehensive analysis of other risk factors related to cardiac tamponade. The patient had been experiencing palpitations prior to receiving acupuncture treatment, which is known to be common symptoms of cardiac tamponade itself (8), and this report cannot reveal any perforation of the heart chamber or the coronary artery after acupuncture treatment. Therefore, it is possible that the patient might have had an underlying cardiac condition before the acupuncture session. This possibility is further substantiated by the echocardiographic finding of an unknown soft tissue in the pericardium, which could indicate pre-existing pathology. While the report states that there is no history of cardiovascular disease, trauma, or surgical interventions, it is important to consider the potential for other underlying causes or the occurrence of spontaneous cardiac tamponade (9, 10). As evidenced by previous case reports, cardiac tamponade can occur unexpectedly due to causes such as blunt trauma, or even spontaneously without any identifiable cause (10, 11). Therefore, it is essential for the authors to include a thorough examination of other potential risk factors.

Finally, this report does not provide detailed information about the acupuncture procedure (i.e., needle length, diameter, stimulation method, and type of practitioner). Without this information, a precise evaluation of the possibility remains unattainable. Although obtaining detailed information about acupuncture is realistically difficult, efforts for gathering such details are still necessary to prove causality. The acupuncture needle has a diameter of 0.2–0.3 mm, unlike pericardiocentesis needles, which used to be 1.2–1.6 mm in the past. Even if it actually penetrates the heart and the following perforation occurs, it would be difficult to rationalize the hemopericardium of 450 ml in 8 h. Furthermore, information about the type of practitioner should be stated, because a qualified practitioner would possess the necessary anatomical knowledge to avoid cardiac structures. Several previous studies have reported cases of hemopericardium caused by acupuncture; however as in this case, the specific procedural details of the treatment were not adequately documented (12, 13). Thus, to ensure accurate assessments of acupuncture-related adverse events, the Standards for Reporting Interventions in Clinical Trials of Acupuncture (STRICTA) guidelines and recommendation for case reports on acupuncture-related traumatic adverse events would be advisory for yielding better reports in future (14, 15).

In conclusion, this report describes a rare case of hemopericardium potentially associated with acupuncture. However, it is a methodologically insufficient report and several additional factors must be considered to establish a definitive causal relationship between acupuncture treatment and this adverse event. In addition, we can assert that fatal adverse events, including the hemopericardium, can be effectively prevented when acupuncture is performed appropriately by licensed and experienced professionals. According to WHO guidelines, it seems that traditional acupuncture practitioners require at least two years of training (2,500 h), including 1,000 h of practical and clinical work, while qualified physicians may need a shorter, focused program (1,500 h) at least. The training must cover core topics such as acupuncture theory, acupuncture techniques, anatomy, safety protocols, and emergency management (16).
